# Targeted Isolation of Lignans from *Trachelospermum asiaticum* Using Molecular Networking and Hierarchical Clustering Analysis

**DOI:** 10.3390/biom10030378

**Published:** 2020-03-01

**Authors:** Jiho Lee, Hong Seok Yang, Hyogeun Jeong, Jung-Hwan Kim, Heejung Yang

**Affiliations:** 1 Laboratory of Natural Products Chemistry, College of Pharmacy, Kangwon National University, Chuncheon 24341, Korea; jiho3232@kangwon.ac.kr (J.L.); comboy10@naver.com (H.S.Y.); jung99gs@naver.com (H.J.); 2 Department of Pharmacology, College of Medicine, Institute of Health Sciences, Gyeongsang National University, Jinju 52727, Korea; junghwan.kim@gnu.ac.kr

**Keywords:** lignans, *Trachelospermum asiaticum*, GNPS, targeted isolation

## Abstract

High-resolution-mass-spectrometry (HR-MS) methods rapidly provide extensive structural information for the isolation of metabolites in natural products. However, they may occasionally provide more information than required and interfere with the targeted analysis of natural products. In this study, we aimed to selectively isolate lignans from *Trachelospermum asiaticum* by applying the Global Natural Product Social Molecular Networking (GNPS) platform and hierarchical clustering analysis (HCA). *T. asiaticum*, which contains lignans, triterpenoids and flavonoids that possess various biological activities, was analyzed in a data-dependent acquisition (DDA) analysis mode using HR-MS. The preprocessed MS spectra were applied not only to GNPS for molecular networking but also to HCA based on similarity patterns between two nodes. The combination of these two methods reliably helped in the targeted isolation of lignan-type metabolites, which are expected to possess potent anti-cancer or anti-inflammatory activities.

## 1. Introduction

*Trachelospermum asiaticum* (Korean name: “Nagseogdeung”, Apocynaceae), which is regionally distributed in East Asian countries such as Korea, China, and Japan, has been reported to contain lignans [1], triterpenoids [2] and flavonoids [3]. As such, it has been used in traditional medicine for treating hypertension and neuralgia. *T. asiaticum* has been reported to exert tuberculosis and bronchitis effects, and has also been used for the treatment of rheumatism [4].

**Figure 1:**
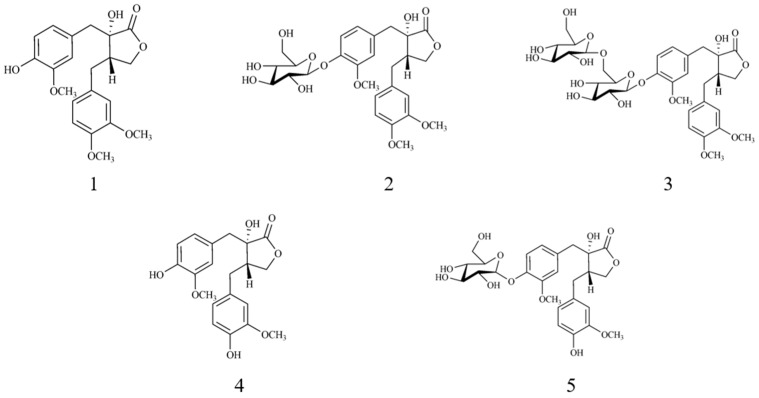
Chemical structures of compounds **1**–**5**.

High-resolution-mass-spectrometry (HR-MS), which employs various instruments such as a quadrupole time-of-flight (qTOF) mass spectrometer and Orbitrap, has become one of the most powerful techniques to obtain information on metabolites in natural products. HR-MS is most helpful for the identification, over many decades, of compounds from natural products [5,6,7]. The data-dependent analysis (DDA) mode of the tandem MS technique using HR-MS detects ions in two stages. It detects two or three ions of the most intense ions in the first stage (MS) and their fragmented ions (MS/MS) in the second stage. The two layers of MS data consisting of the parent ions and their fragmented ions are very useful for annotating unidentified peaks. Recently, the Global Natural Product Social (GNPS) platform has received increasing attention among natural product chemists [8]. GNPS helps to process numerous *m/z* and intensity values from raw MS spectral data acquired in the DDA mode. The GNPS also helps generate molecular networks (MNs) based on the similarity between processed MS spectra of single compounds. Nevertheless, MN only focuses on the structural similarity between two nodes. In the present study, we applied hierarchical clustering analysis (HCA) to obtain insights into the targeted isolation of lignans from nodes that are not directly connected by an MN but have the same chemical scaffolds. The combination of molecular networking and HCA was more reliable than the application of each method alone for the targeted isolation of five lignans (**1**–**5**) from *T. asiaticum* roots (Figure 1). We could isolate compounds **1**–**5** which have cytotoxic activities against four cell lines, namely, human lung cancer (A549), ovarian cancer (SKOV3), prostate cancer (PC3) and laryngeal carcinoma (Hep2) cells.

## 2. Results and Discussion

### 2.1. Molecular Networking and Hierarchical Clustering Analysis of Mass Spectral Data from T. asiaticum

**Figure 2:**
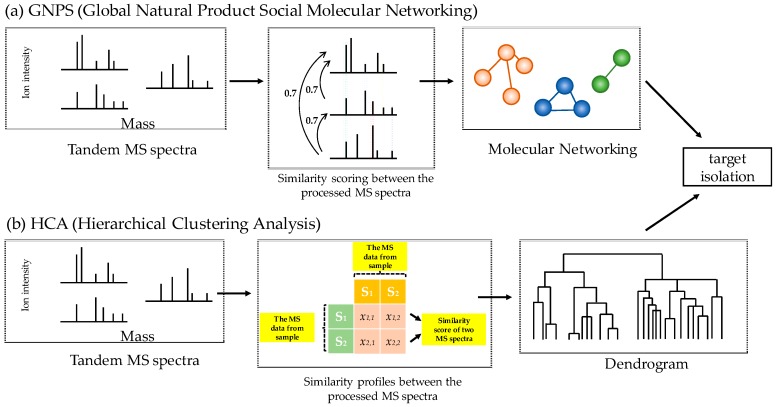
Workflow of molecular networking and hierarchical clustering analysis (HCA). MS spectral data from *Trachelospermum asiaticum* were acquired by high-resolution mass spectrometry (See Materials and Methods section) and preprocessed by the MZmine software (Version. 2.34). The molecular network was constructed using the Global Natural Product Social Molecular Networking (GNPS) platform. The processed MS spectra with similar patterns (cosine similarity >0.7) were grouped into clusters (**a**). The matrix profile was generated using similarity scores between the processed MS spectra which were calculated by the Pearson correlation coefficient and visualized in a dendrogram (**b**).

In the present study, we compared the HCA results between the structural similarities of the processed MS spectral data derived from single compounds with the molecular networking results obtained through GNPS (Figure 2). The molecular networking results showed connections between two nodes with similar spectral patterns (cosine similarity threshold >0.7). The molecular type of the cluster was identified by comparison with MS databases [8]. We attempted to selectively isolate specific types of compounds using the information on the nodes annotated by the MN. Although these nodes were derived from the same backbone, the different strong fragmented ions lowered the similarity score and hindered the connection between them. We applied HCA to improve the results of molecular networking. The matrix profile generated using similarity scores provided clear evidence on the nodes that were absent in the MN and the results were presented as a dendrogram [9]. The nodes with similar score profiles were more closely located in the smaller clusters.

**Figure 3:**
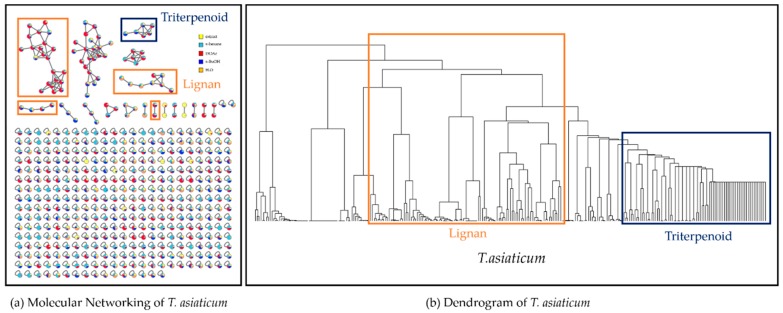
Molecular networking of *Trachelospermum asiaticum* using the GNPS platform (**a**) and Dendrogram of HCA results (**b**).

*Trachelospermum* species contains dibenzylbutylrolactone-type lignans as a bioactive component. These lignans possess potent anti-estrogenic [10], antitumor [11] and anti-cancer activities [12]. In the present study, we focused on the isolation of dibenzylbutylrolactone-type lignans from the methanolic extract of *T. asiaticum* using two approaches, GNPS and HCA (Figure 3). The MN from the total methanolic extract and the four sub-fractions of *T. asiaticucm*, *n*-hexane, EtOAc, *n*-BuOH and H_2_O, comprised 489 nodes, with 144 paired nodes and 345 non-cluster nodes. Based on the node information provided by the network annotation propagation (NAP) tool, an in silico node annotation tool, we could annotate the nodes from the total extract and four sub-fractions for the discovery of dibenzylbutylrolactone-type lignans using the MN (Figure 3a) (See Figure S6 in Supplementary Materials). In the MN, the nodes annotated as dibenzylbutylrolactone-type lignans mainly existed in the E6 sub-fraction from the EtOAc and the B3 sub-fraction from the *n*-BuOH sub-fractions, respectively (See Figures S7 and S8 in Supplementary Materials). Next, we found that the HCA results showed that the nodes for lignans and triterpenoids, which were not grouped together in the sub-cluster of the MN, were closely gathered together by the same backbones and distinctly separated by different ones (Figure 3b). Based on these two in silico results, we aimed for targeted isolation using the information on the nodes annotated as dibenzylbutylrolactone-type lignans. Consequently, we successfully isolated five dibenzylbutylrolactone-type lignans from *T. asiaticum* roots.

### 2.2. Targeted Isolation of Compounds ***1**–**5*** Using Molecular Networking and HCA Results

**Figure 4:**
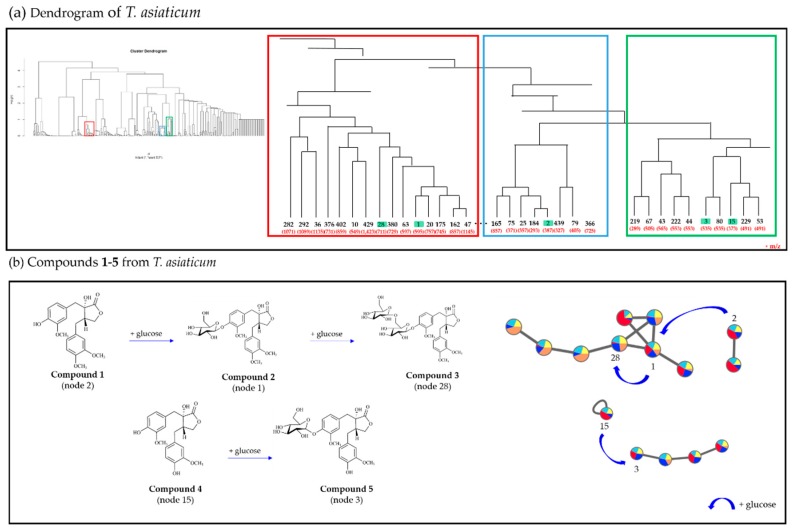
Compounds **1**–**5** identified based on molecular networking and HCA results.

The five lignans were identified as trachelogenin (**1**) [13], tracheloside (**2**) [14], trachelogenin *β*-gentionbioside (**3**) [15], nortrachelogenin (**4**) [16] and nortracheloside (**5**) [17] by comparing them with previously reported spectroscopic data. Nodes from compounds **1**–**5** were easily displayed in the molecular networking and HCA results (Figure 4). Notably, in the MN, nodes 2 (*m/z* 387.14 [M−H]^−^), 1 (*m/z* 595.20 [M+Glc+2Na]^−^) and 28 (*m/z* 711.24 [M+2Glc]^−^) for compounds **1–3**, which have the same backbone except for the sequential attachment of glucose moieties, were not present in one cluster but they were closely located in the HCA results (Figure 4).

Compound **5** had an additional glucose unit compared to compound **4**; however, compounds **4** (node 15) and **5** (node 3) were not clustered in the same sub-group in the MN. Contrastingly, nodes 3 and 15 in the HCA results were closely located together in the low-level cluster. It was confirmed that node 114 possesses an additional glucose compared to compound **5** and was identified as nortrachelogenin 4,4′-di-*O*-*β*-d-glucopyranoside [18,19]. Node 114, which was not clustered in GNPS, was found near compounds **4** and **5** in the HCA (See Figure S8 in Supplementary Materials).

In the present study, we found that some nodes (e.g., 15, 114, and 141) that were not connected in GNPS could be located in close clusters in the dendrogram. This approach helped to predict the structures corresponding to unknown nodes. Nodes 21 and 69, which were included in the same cluster in the MN and closely located in the dendrogram, were predicted as dibenzylbutylrolactone-type lignans. Node 21 with an *m/z* value of 519.19 [M+Glc]¯ was proposed as the matairescinoside [20]. The MS spectral pattern corresponding to node 69 (*m/z* 682.24 [M+2Glc]¯) showed one more glucose moiety attached to the compound at node 21, and the compound was suggested as matairesinol 4,40-di-*O*-*β*-d-glucopyranoside [2] (See Figure S9 in Supplementary Materials).

Recently, the GNPS platform has become one of the best choices for natural product chemists to discover novel chemicals from natural products and to determine their metabolic changes. Molecular networking using a GNPS platform can help to visualize the connectivity between similar structures. Because the connectivity of nodes in the MN can only be decided by similarity scoring between every two MS spectra, the MN occasionally failed to identify the clustering of the same types of backbone. In the present study, we found that HCA, which compares a single node against all the nodes, provided clearer results for the annotation of backbones than molecular networking. By combining the results of molecular networking and HCA, we could easily identify and selectively isolate dibenzylbutylrolactone-type lignans from the extract of *T. asiaticum* roots.

### 2.3. Cytotoxic Activity of Isolated Compounds ***1**–**5***

The cytotoxic activities of compounds **1**–**5** were tested against four cell lines (A549, SKOV3, PC3 and hep2) using a 3-(4,5-Dimethylthiazol-2-yl)-2,5-diphenyltetrazolium bromide (MTT) assay (Table 1). Compounds **1**–**5** exhibited different cytotoxic activities against different cell lines. Compounds **1** and **4** showed cytotoxic activities against the A549 cancer cell line with IC_50_ values of 19.5 and 20.6 μM, respectively. Compounds **2**, **4,** and **5** exhibited cytotoxicity against the SKOV3 cell line with IC_50_ values of 23.8, 24.7 and 23.2 μM, respectively. Compound **1** exhibited cytotoxicity against HEP2 cells with an IC_50_ value of 18.3 μM, while compound **2** showed cytotoxicity against the PC3 cell line with an IC_50_ value of 19.3 μM.

## 3. Materials and Methods

### 3.1. Plant Material

The roots of *T. asiaticum* were collected from Jinju, Korea in May 2018 and deposited in the Herbarium at the College of Pharmacy, Kangwon National University (KNUTA-01).

### 3.2. Apparatus and Reagents

^1^H NMR data were recorded at 600 MHz on a Bruker Avance Neo 600 (Brucker, Billerica, MA, USA) spectrometer in the Central Laboratory of Kangwon National University (Chuncheon, Korea). The Mass experiements were performed on a Waters Xevo G2 qTOF mass spectrometer (Waters MS Technologies, Manchester, UK) with the UPLC system through an electrospray ionization (ESI) interface for the MN study, as well as the exact mass analysis. Silica gel Kieselgel 60 (40–60 μm, 230–400 mesh, Art. 9385, Merck, Darmstadt, Land Hessen, Germany) and Diaion HP-20 (Mitsubishi Chemical Industries Ltd., Chiyoda-ku, Tokyo, Japan) were used for column chromatography. Thin layer chromatography (TLC) was performed to monitor the different fractions of *T. asiaticum* using a Kieselgel 60 F_254_ (Art. 5715, Merck, Darmstadt, land Hessen, Germany) and an RP-C_18_ F_254_ (Art. 15389, Merck, Darmstadt, Land Hessen, Germany). Semipreparative HPLC was performed on an Agilent 1260 Infinity Quaternary LC (Agilent, Santa, CA, USA) with an Inspire^TM^ 5 μm C_18_ (250 × 21.2 mm, Dikima, Foothill Ranch, CA, USA). Methanol (MeOH), *n*-hexane, ethyl acetate (EtOAc) and *n*-butanol (*n*-BuOH) were purchased from Daejung (Si-heung, Korea). The other reagents were purchased from Sigma-Aldrich (St. Louis, MO, USA).

### 3.3. Extraction and Isolation

The dried roots of *T*. *asiaticum* (696.8 g) were extracted with 100% MeOH (each for 3 days) at room temperature. The methanol extract (71.7 g) was fractionated with *n*-hexane (3.0 g), EtOAc (6.5 g), *n*-BuOH (24.8 g) and H_2_O (33.4 g), successively. The *n*-BuOH (24.8 g) fraction of *T. asiaticum* was applied to a HP-20 resin column, eluted with MeOH-H_2_O (0:1, 2:3, 1:1, 3:2 and 1:0) to yield five fractions (B1: 2.9 g, B2: 1.6 g, B3: 5.7 g, B4: 2.6 g, B5: 3.5 g). The fraction B3 (5.7 g) was re-chromatographed by reversed-phase medium-pressure liquid chromatography (MPLC) silica gel with MeOH-H_2_O (1:4, 1:3, 1:2, 1:1, 2:1, and 1:0) to yield seven fractions (B3-1 to 7), which were collected and monitored by TLC analysis. Fraction B3-5 was applied to reversed-phase MPLC silica gel eluted with MeOH-H_2_O (1:4, 1:3, 1:2, 1:1, 2:1, and 1:0) to afford six fractions. Fraction B3-5-4 (374.2 mg) was applied to a preparative HPLC [mobile phase MeCN-H_2_O with 0.1% formic acid (60:40)] to produce compounds **3** (18 mg) and **5** (128.2 mg).

The EtOAc (6.5 g) fraction of *T. asiaticum* was separated by MPLC silica gel with n-hexane and ethyl acetate (HE) (5:1, 1:1, 1:5), chloroform and methanol (CM) (5:1, 3:2, 2:3, 1:5), and MeOH 100% to yield compound **2** (1.8 g) and 12 fractions (E1 ~ 12). These fractions were monitored by TLC analysis. E6 (357.1 mg) was subjected to preparative HPLC [mobile phase MeCN-H_2_O with 0.1% formic acid (50:50)] to produce compounds **1** (94.6 mg) and **4** (25 mg).

#### 3.3.1. Trachelogenin (**1**)

Yellow gum, [*α*]D − 36° (*c* = 0.43, CHCl_3_); HR-MS *m/z:* 387.1480 [M−H]^−^ (calcd for C_21_H_23_O_7,_ 387.1444); ^1^H NMR (600 MHz, CDCl_3_) *δ*_H_: 2.51 (m, 1H, H-8), 2.54 (m, 1H, H-7), 2.93 (d, *J* = 5.0 Hz, 1H, H-7), 2.96 (m, 1H, H-7′), 3.11 (d, *J* = 13.7 Hz, 1H, H-7′), 3.84, 3.85, 3.86 (s, 3H each, -OMe), 4.00 (m, 1H, H-9), 4.04 (m, 1H, H-9), 6.62 (d, 1H, *J* = 2.0 Hz, H-2), 6.63 (d, 1H, *J* = 1.1 Hz, H-6′), 6.67 (dd, *J* = 8.1, 1.9 Hz, 1H, H-6), 6.71 (d, *J* = 1.9 Hz, 1H, H-2′), 6.79 (d, 1H, *J* = 8.1 Hz, H-5), 6.84 (d, 1H, *J* = 8.0 Hz, H-5′).

#### 3.3.2. Tracheloside (**2**)

Amorphous powder, ${\left[\alpha \right]}_{D}^{27}$ − 12.6° (*c* = 1.6, MeOH); HR-MS *m/z*: 549.1970 [M−H]^−^ (calcd for C_27_H_33_O_12_, 549.1972); ^1^H NMR (600 MHz, pyridine-*d*_5_) *δ*_H_: 2.77 (ddd, *J* = 17.3, 9.5, 5.0 Hz, 1H, H-8), 2.96 (dd, *J* = 13.8, 10.0 Hz, 1H, H-7), 3.26 (dd, *J* = 13.8, 4.8 Hz, 1H, H-7), 3.34 (d, *J* = 13.6 Hz, 1H, H-7′), 3.66 (d, *J* = 13.6 Hz, 1H, H-7′), 3.76, 3.77, 3.82 (s, 3H each, -OMe), 4.19 (t, *J* = 8.0 Hz, 1H, H-9), 4.36 (m, 1H, H-9), 5.67 (d, *J* = 6.2 Hz, 1H, H_glc_-1), 6.89 (dd, *J* = 8.1, 1.6 Hz, 1H, H-6), 6.93 (s, 1H, H-2), 6.94 (d, *J* = 7.6 Hz, 1H, H-5), 7.06 (dd, *J* = 8.3, 1.7 Hz, 1H, H-6′), 7.17 (d, *J* = 1.7 Hz, 1H, H-2′), 7.60 (d, *J* = 8.3 Hz, 1H, H-5′).

#### 3.3.3. Trachelogenin β-Gentionbioside (**3**)

Amorphous powder, ${\left[\alpha \right]}_{D}^{28}$ − 70.0° (*c* = 2.0, MeOH); HR-MS *m/z:* 711.2489 [M−H]^−^ (calcd for C_33_H_43_O_17_, 711.2500); ^1^H NMR (600 MHz, pyridine-*d*_5_) *δ*_H_: 2.76 (ddd, *J* = 17.3, 9.4, 5.1 Hz, 1H, H-8), 2.93 (dd, *J* = 13.8, 10.1 Hz, 1H, H-7), 3.22 (dd, *J* = 13.9, 7.0 Hz, 1H, H-7), 3.30 (d, *J* = 13.6 Hz, 1H, H-7′), 3.61 (d, *J* = 13.9 Hz, 1H, H-7′), 3.75, 3.76, 3.81 (s, 3H each, -OMe), 3.85 (m, 1H, H-8), 4.04 (t, *J* = 8.0 Hz, 1H, H-9), 4.49 (dd, *J* = 11.8, 2.2 Hz, 1H, H-9), 5.06 (d, *J* = 7.8 Hz, 1H, H_glc_-1′), 5.58 (d, *J* = 7.3 Hz, 1H, H_glc_-1), 6.88 (dd, *J* = 8.1, 1.6 Hz, 1H, H-6), 6.92 (d, *J* = 1.6 Hz, 1H, H-2), 6.94 (d, *J* = 8.1 Hz, 1H, H-5), 7.14 (d, *J* = 1.7 Hz, 1H, H-2′), 7.19 (d, *J* = 1.6 Hz, 1H, H-6′), 7.70 (d, *J* = 8.3 Hz, 1H, H-5′).

#### 3.3.4. Nortrachelogenin (**4**)

Yellow resin, [*α*]D + 15.4° (*c* = 0.52, CHCl_3_); HR-MS *m/z:* 373.1307 [M−H]^−^ (calcd for C_20_H_21_O_7_, 373.1287); ^1^H NMR (600 MHz, CDCl_3_) *δ*_H_: 2.49 (m, 1H, H-8), 2.53 (m, 1H, H-7), 2.92 (d, *J* = 4.1 Hz, 1H, H-7), 2.93 (d, *J* = 7.1 Hz, 1H, H-7′), 3.12 (d, *J* = 13.7 Hz, 1H, H-7′), 3.84, 3.82 (s, 3H each, -OMe), 3.98 (dd, *J* = 15.6, 6.8 Hz, 1H, H-9), 4.03 (m, 1H, H-9), 6.60 (s, 1H, H-5′), 6.61 (s, 1H, H-2′), 6.63 (m, 1H, H-5), 6.70 (d, *J* = 1.3 Hz, 1H, H-2), 6.82 (s, 1H, H-6′), 6.83 (d, *J* = 3.7 Hz, 1H, H-6).

#### 3.3.5. Nortracheloside (**5**)

White powder, ${\left[\alpha \right]}_{D}^{19}$ − 47.9° (*c* = 1.02, EtOH); HR-MS *m/z:* 534.1850 [M−H]^−^ (calcd for C_26_H_31_O_12,_ 535.1816); ^1^H NMR (600 MHz, DMSO-*d_6_*) *δ*_H_: 2.38 (m, 1H, H-8), 2.45 (dd, *J* = 13.6, 9.9 Hz, 1H, H-7), 2.69 (dd, *J* = 14.7, 4.4 Hz, 1H, H-7), 2.91 (d, *J* = 13.5 Hz, 1H, H-7′), 3.09 (d, *J* = 13.5 Hz, 1H, H-7′), 3.71, 3.73 (s, 3H each, -OMe), 3.97 (d, *J* = 7.7 Hz, 2H, H-9), 4.93 (d, *J* = 7.3 Hz, 1H, H_glc_-1′), 6.56 (dd, *J* = 8.0, 1.6 Hz, 1H, H-5′), 6.69 (d, *J* = 1.7 Hz, 1H, H-6′), 6.72 (d, *J* = 8.0 Hz, 1H, H-2′), 6.75 (dd, *J* = 8.4, 1.5 Hz, 1H, H-5), 6.81 (d, *J* = 1.6 Hz, 1H, H-6), 7.05 (d, *J* = 8.4 Hz, 1H, H-2).

### 3.4. GNPS Analysis

The raw data of total methanolic extract and four sub-fractions from *T.* *asiaticum*, *n*-hexane, EtOAc, *n*-BuOH and H_2_O, were acquired by performing UPLC-MS/MS. The mobile phases involved a mixture with H_2_O (A) buffered with 0.1% formic acid and acetonitrile (B). 5%–95% B (0–13 min), 95% B (13–14.5 min), 95%–5% B (14.5–14.7 min) and 5% B (14.7–15 min). The flow rate was set at 300 μL/min. The temperatures in the autosampler and in the column oven were set at 10 and 45 °C, respectively. The ESI conditions for MS analyses were set as follows: negative ion mode, capillary voltage of 2.5 kV, cone voltage of 20 V, source temperature of 120 °C, desolvation temperature of 350 °C and a desolvation gas flow of 800 L/h. The ion acquisition rate was 0.3 s with a resolution in excess of 20,000 FWHM. The energy for the collision-induced dissociation (CID) was set to 4 V for the precursor ions in the MS1 scan, and MS/MS scans were acquired in negative ion automated data-dependent acquisition (DDA) mode, in which MS/MS scans for the three most intense ion were produced (scan time 100 ms). The MS/MS acquisition was set to be activated when the Total Ion Current (TIC) of the MS1 survey scan rose and switched back to survey scanning after two scans of MS/MS. The MS1 and MS/MS data were converted to XML format by MZmine software (Ver. 2.3.4). The MN was constructed using the website GNPS (https://gnps.ucsd.edu/ProteoSAFe/static/gnps-splash.jsp). The parent mass tolerance was 0.05 Da and the MS/MS fragment ion tolerance was set to 0.05 Da. Subsequently, consensus spectra containing less than three spectra were eliminated. The MN was created using a cosine score above 0.7 and more than three matched peaks. The mass spectral data in the MN were explored by comparing with the GNPS spectral libraries. The MN was visualized with Cytoscape 3.7.0 (http://www.cytoscape.org/) and the information on the nodes and edges in the MN were found at a GNPS repository (GNPS project ID: a81b6ca055a64428b42ca7971bac677b).

### 3.5. Dendrogram Analysis

HCA was created with R program version (3.6.1) using “dendextend” and “qplots” packages, which indicate the hierarchical similarity between mass data.

### 3.6. Cytotoxicity Assay

Compounds **1**–**5** were tested for their cytotoxic activity against A549, SKOV3, PC3 and Hep2 cells using an MTT assay. Etoposide was used as a positive control. A549, SKOV3, PC3, and Hep2 cells were seeded at a density of 5 × 10^3^ cells/well in a 96-well plate. After overnight incubation, compounds **1**–**5** were dissolved in dimethyl sulfoxide and treated with different concentrations (10–100 μM). After 48 h of incubation in a 37 °C incubator, cell viability was evaluated at 490 nm. IC_50_ values were calculated as the mean of three-times treatment tests.

## 4. Conclusions

We constructed an MN of total methanolic extract fraction and sub-fractions (*n*-hexane, EtOAc, *n*-BuOH and H_2_O) from *T. asiaticum* using the GNPS platform. In our study, some clusters in the MN included nodes annotated as lignans and triterpenoids, but many other nodes that were not connected in the cluster were not annotated. To overcome the general limitation of GNPS, we used the similarity score profiles of the MS spectral data of every single node against the similarity score profiles corresponding to all of the nodes. The nodes that had more similar backbones were closely located together in the lower branches. Using this proof of concept, we could identify the nodes for lignans and triterpenoids in the dendrogram. Some nodes that were not grouped in the MN were located in the lower branches of the dendrogram and were successfully utilized for the targeted isolation of five dibenzylbutylrolactone-type lignans (**1**–**5**). Thus, the combination of molecular networking and HCA improved the experimental efficiency of the targeted isolation of compounds possessing cytotoxic activities against four cancer cell lines. In the next study, we will try to automatically compare the MN and HCA results for the simple and rapid annotation of nodes.

## Figures and Tables

**Table 1:** Cytotoxicity data of compounds **1**–**5** from *Trachelospermum asiaticum.*

Compound	IC_50_ (μM) ^1^			
	A549	SKOV3	PC3	HEP2
**1**	19.5	63.3	27.8	18.3
**2**	67.9	23.8	19.3	>100
**3**	33.7	72.7	48.7	46.7
**4**	20.6	24.7	55.6	43.1
**5**	46.9	23.2	40.8	47.2
Etoposide ^2^	6.56	6.57	7.78	4.14

^1^ The results are IC_50_ values of compounds against each cancer cell line; ^2^ Etoposide was used as the positive control.

## Supplementary Materials

The following are available online at https://www.mdpi.com/2218-273X/10/3/378/s1: Figures S1–S5: are NMR data of compounds (**1**–**5**); Figure S6: presents the NAP data of *T. asiaticum*; Figure S7: presents the GNPS of B1-B5 fractions; Figure S8: presents the GNPS of E1-E12 fractions; Figures S9 and S10: are GNPS and HCA data of prediction nodes.
