# Supplementary material for: Targeted Isolation of Lignans from Trachelospermum asiaticum Using Molecular Networking and Hierarchical Clustering Analysis
Source: Biomolecules. 2020 Mar 1;10(3):378. doi: 10.3390/biom10030378 (PMC7175116; doi:10.3390/biom10030378)
Supplement: Supplementary file 1 [file biomolecules-10-00378-s001.pdf]

*Article*

# Targeted Isolation of Lignans from *Trachelospermum asiaticum* using Molecular Networking and Hierarchical Clustering Analysis

**Jiho Lee [1], Hong Seok Yang [1], Hyogeun Jeong [1], Jung-Hwan Kim [2] and Heejung Yang [1,*]**

[1] Laboratory of Natural Products Chemistry, College of Pharmacy, Kangwon National University, Chuncheon 24341, Korea; jiho3232@kangwon.ac.kr (J.L.); comboy10@naver.com (H.S.Y.); jung99gs@naver.com (H.J.)
[2] Department of Pharmacology, Gyeongsang National University, Jinju 52727, Korea; junghwan.kim@gnu.ac.kr (J.K)
* Correspondence: heejyang@kangwon.ac.kr; Tel.: +82-33-250-6919

# List of Figures


**Figure S1**. $^1$H NMR spectrum of Trachelogenin (**1**) (600 MHz, $CDCl_3$)

**Figure S2.** $^1$H NMR spectrum of Tracheloside (**2**) (600 MHz, pyridine-$d_5$)

**Figure S3.** $^1$H NMR spectrum of Trachelogenin $\beta$-Gentionioside (**3**) (600 MHz, pyridine-$d_5$)

**Figure S4.** $^1$H NMR spectrum of Nortrachelogenin (**4**) (600 MHz, $CDCl_3$)

**Figure S5.** $^1$H NMR spectrum of Nortracheloside (**5**) (600 MHz, DMSO-$d_6$)

**Figure S6.** NAP results of *Tracheolospermum asiaticum*

**Figure S7.** Molecular Networks (MN) of the sub-fractions of B1~B5

**Figure S8.** Molecular Networks of the sub-fractions of E1~E12

**Figure S9.** The comparison between MN and HCA results of nodes for compounds **4**, **5** and node 114.

**Figure S10.** The **c**omparison between MN and HCA results of nodes 21 and 69

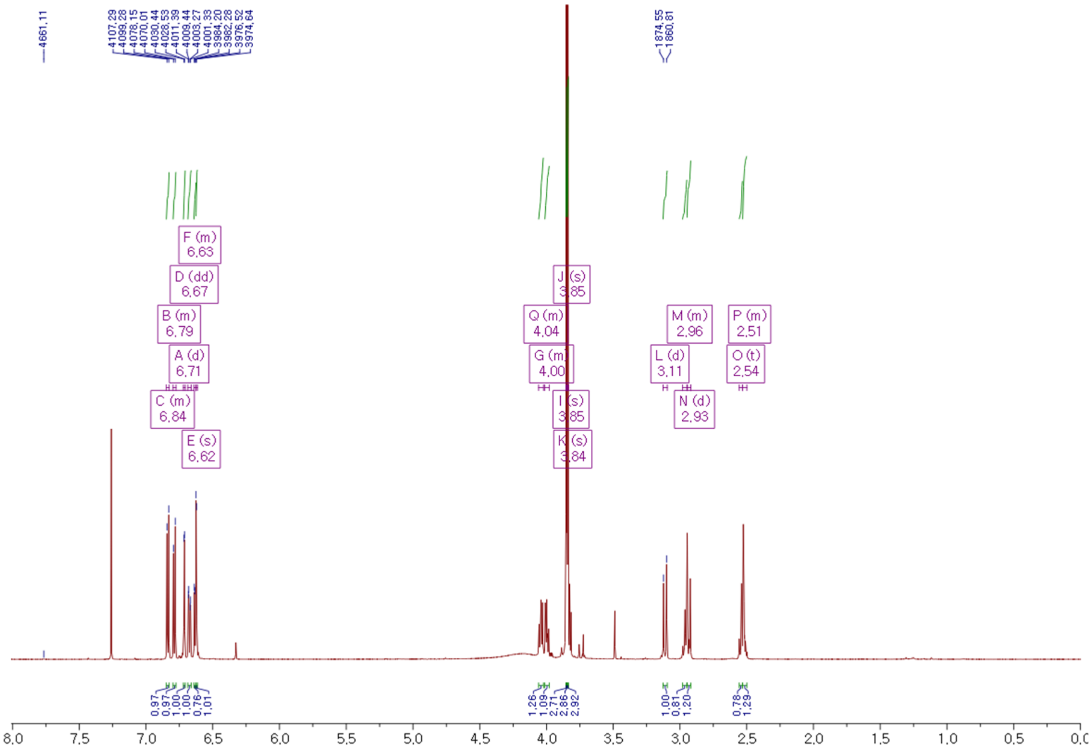

**Figure S1.** $^{1}$H NMR spectrum of Trachelogenin (**1**) (600 MHz, $CDCl_3$).

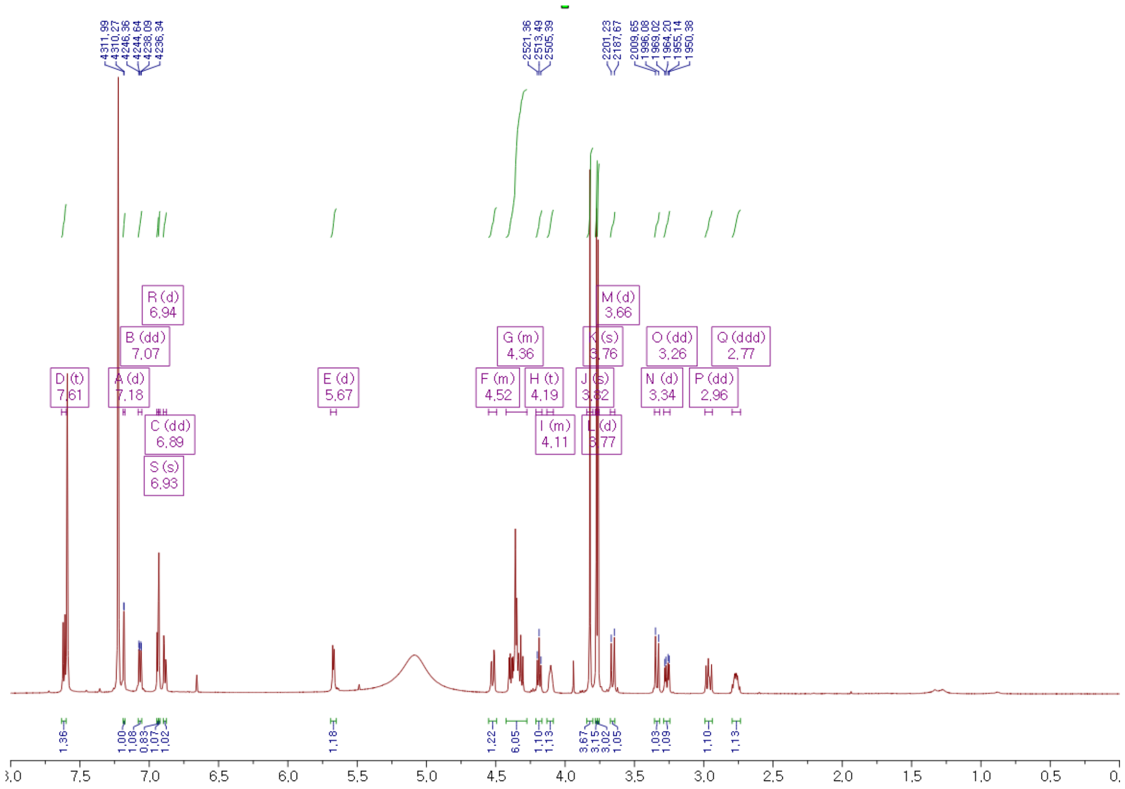


**Figure 2.** $^{1}$H NMR spectrum of Tracheloside (**2**) (600 MHz, pyridine-*d*5).

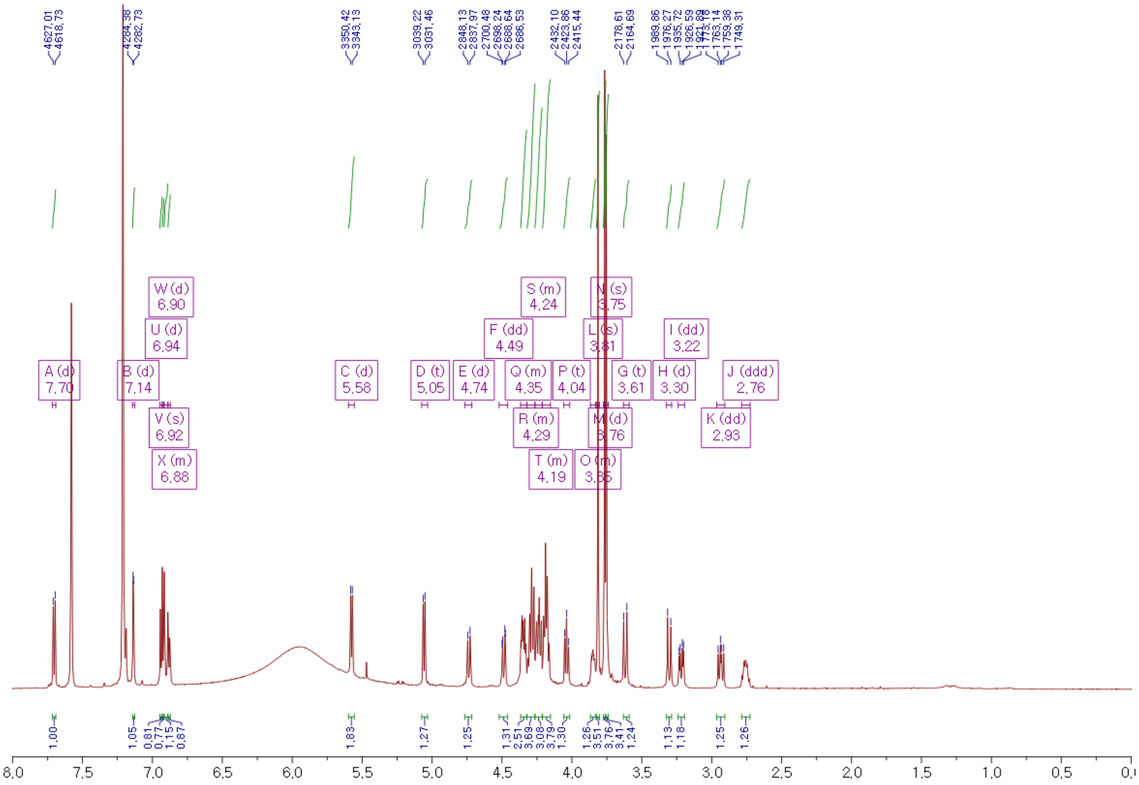

**Figure 3.** $^{1}$H NMR spectrum of Trachelogenin *β*-Gentionioside (**3**) (600 MHz, pyridine-$d_5$).

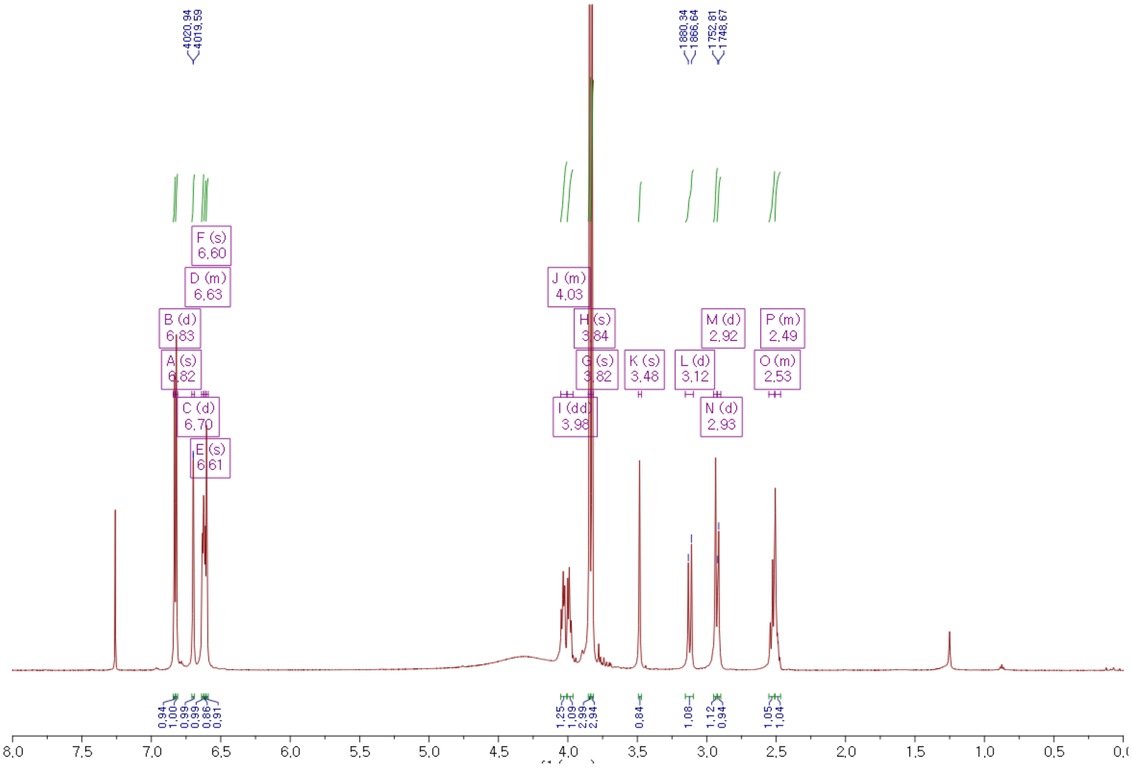


**Figure 4.** $^1$H NMR spectrum of Nortrachelogenin (**4**) (600 MHz, $CDCl_3$).

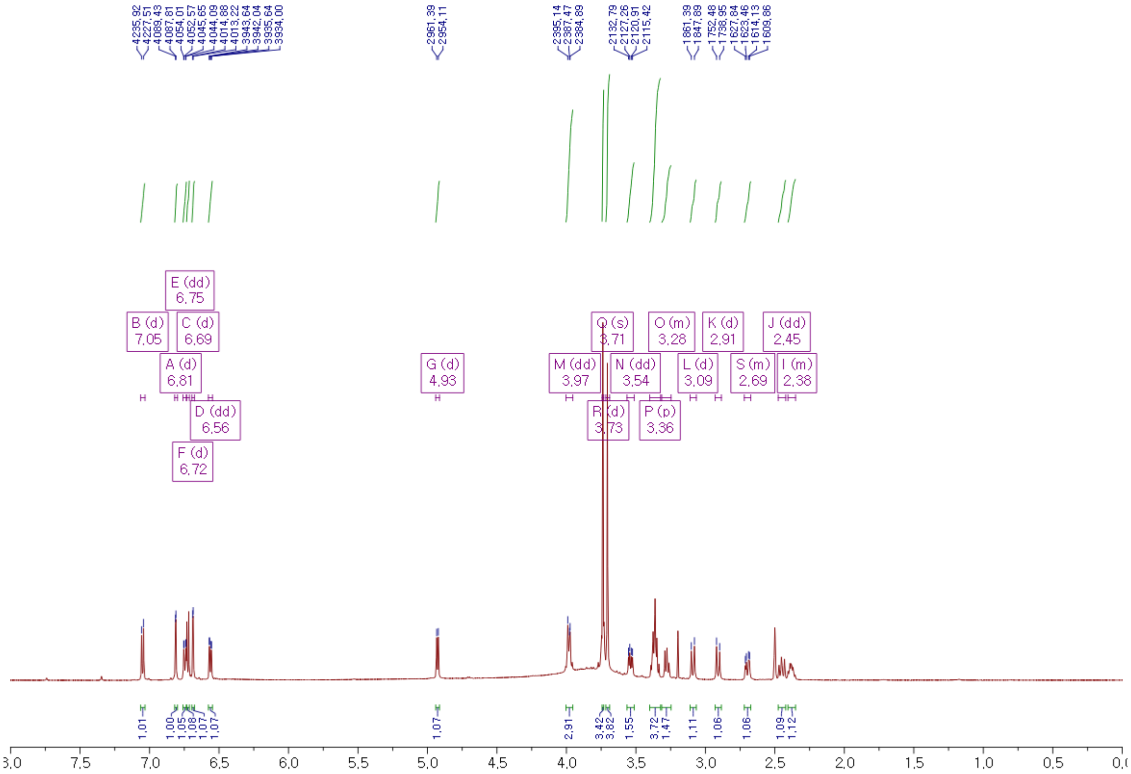

**Figure 5**. $^{1}$H NMR spectrum of Nortracheloside (**5**) (600 MHz, DMSO-$d_6$).

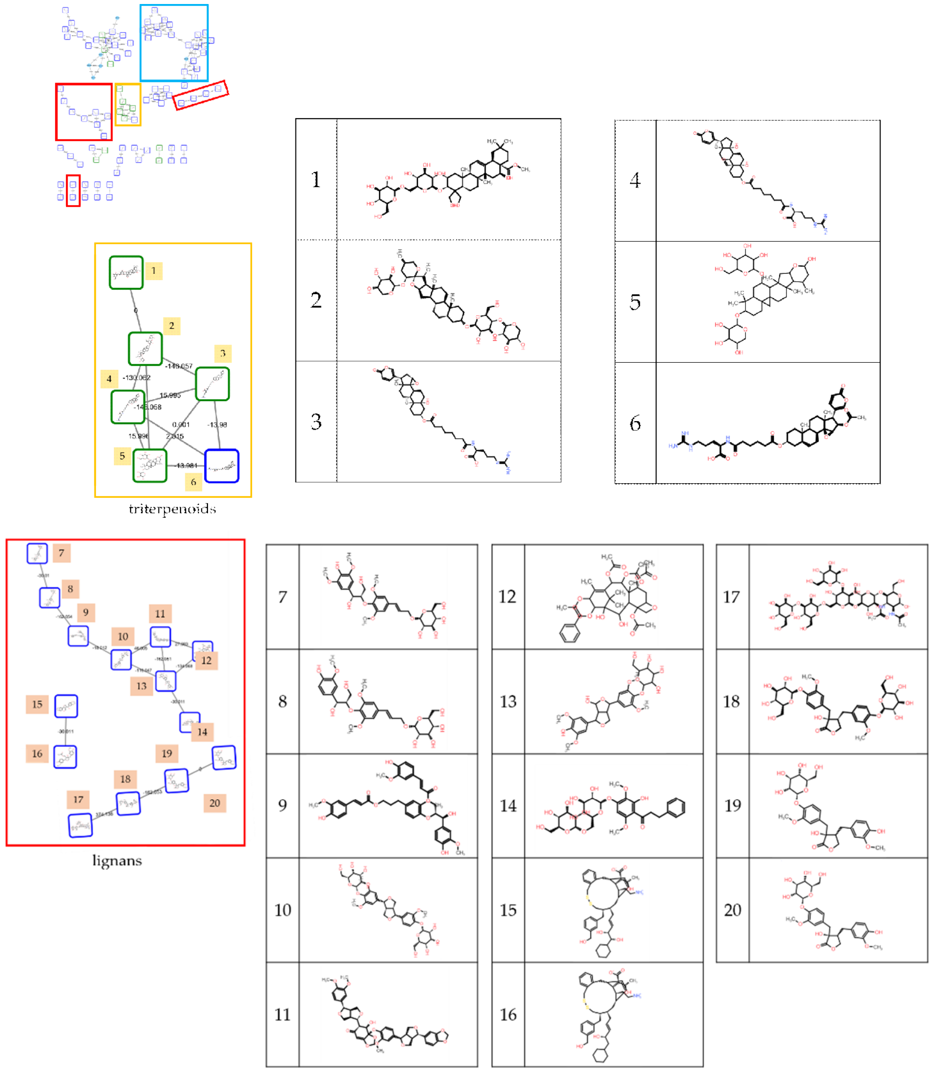
triterpenoids
lignans

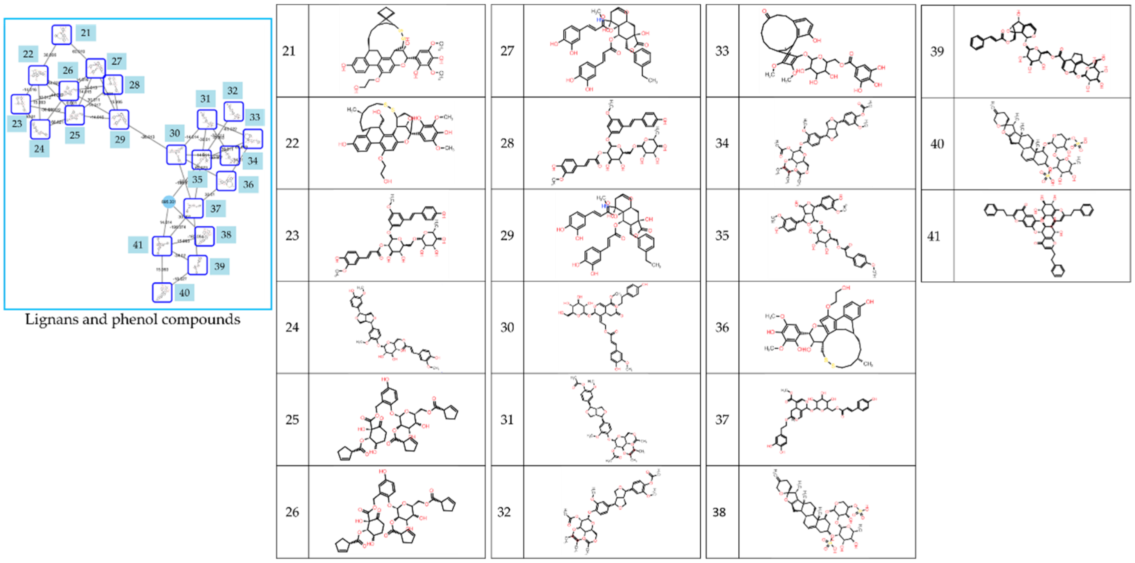


**Figure S6.** NAP results of *Tracheolospermum asiaticum.*

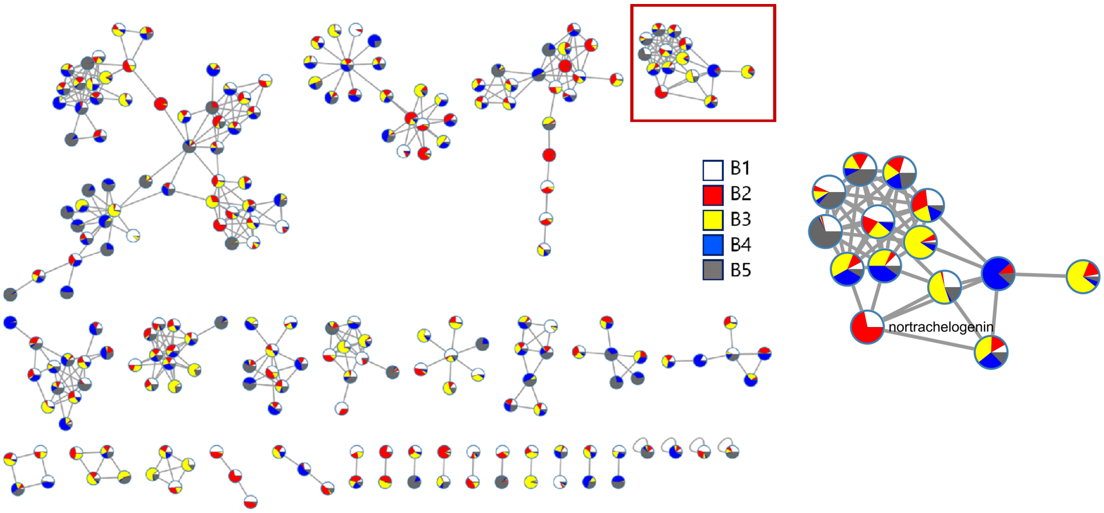


**Figure S7.** Molecular Networks (MN) of the sub-fractions of B1~B5

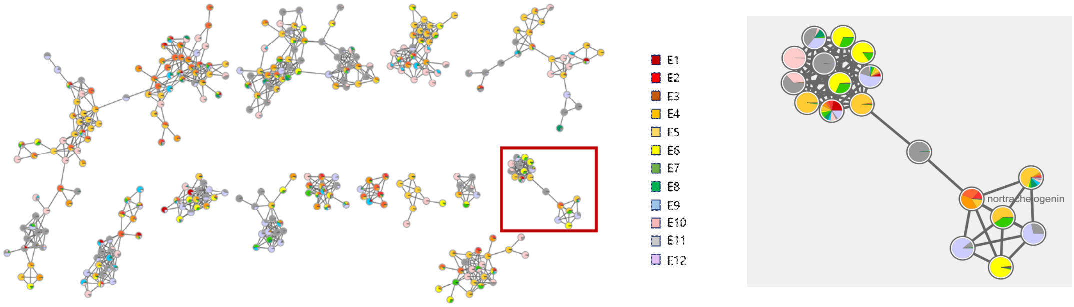


**Figure S8.** Molecular Networks of the sub-fractions of E1~E12

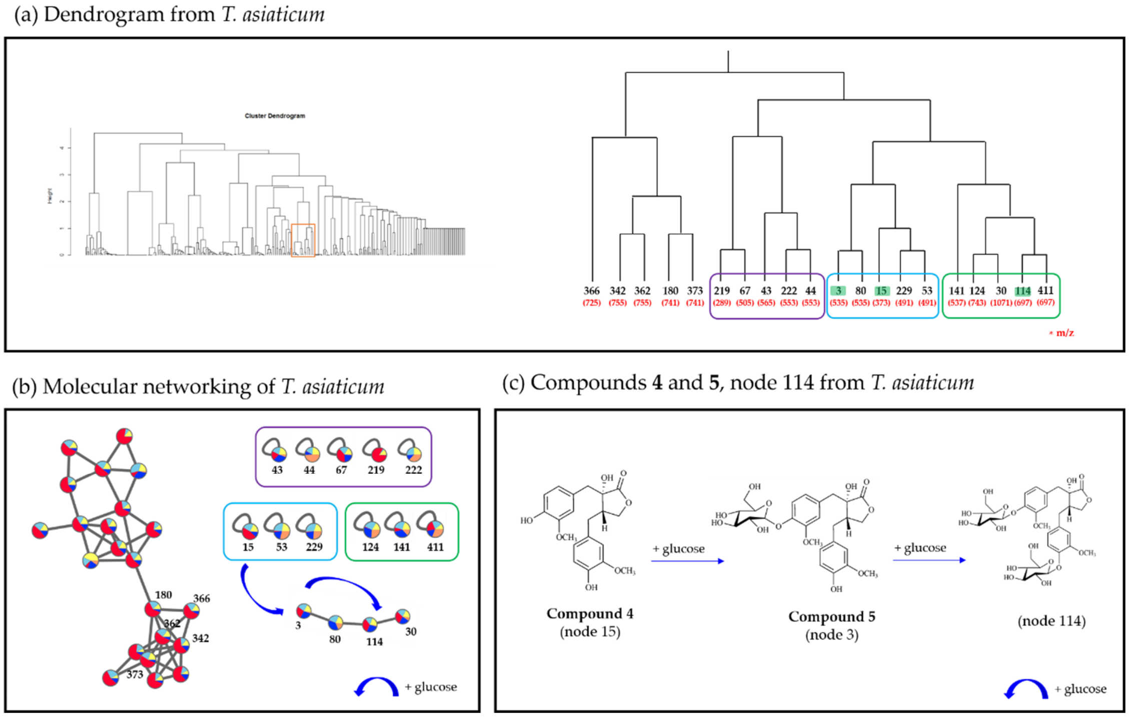


**Figure S9.** The comparison between MN and HCA results of nodes for compounds **4**, **5** and node 114.

.

(a) Dendrogram of *T. asiaticum*

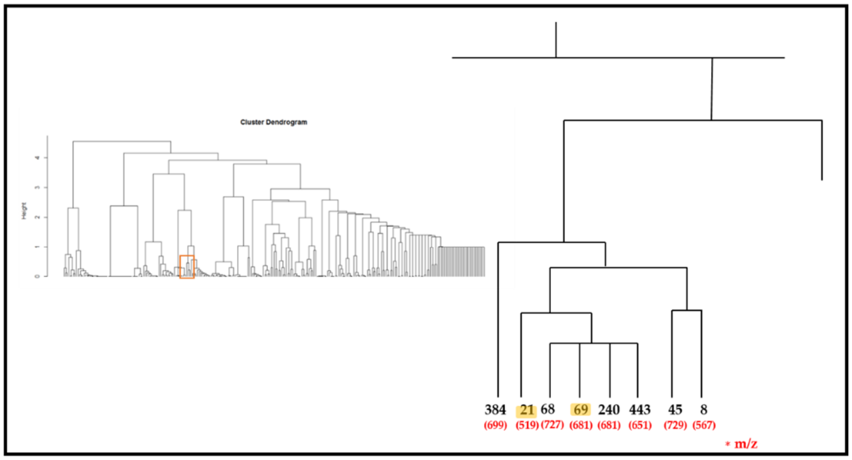


(b) Nodes 21 and 69 from *T. asiaticum*

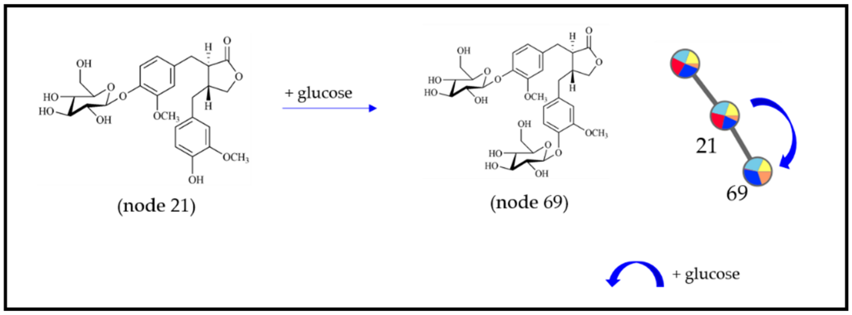


**Figure S10.** The comparison between MN and HCA results of nodes 21 and 69.
